# The role of hepatobiliary scintigraphy combined with spect/ct in predicting severity of liver failure before major hepatectomy: a single-center pilot study

**DOI:** 10.1007/s13304-020-00907-2

**Published:** 2020-11-02

**Authors:** Matteo Serenari, Chiara Bonatti, Lucia Zanoni, Giuliano Peta, Elena Tabacchi, Alessandro Cucchetti, Matteo Ravaioli, Cinzia Pettinato, Alberto Bagni, Antonio Siniscalchi, Antonietta D’Errico, Rita Golfieri, Stefano Fanti, Matteo Cescon

**Affiliations:** 1 General Surgery and Transplantation Unit, Azienda Ospedaliero-Universitaria di Bologna, Sant’Orsola-Malpighi Hospital, Bologna, Italy; 2 Department of Medical and Surgical Sciences, DIMEC, Alma Mater Studiorum, University of Bologna, Via Massarenti 9, 40138 Bologna, Italy; 3 Nuclear Medicine Unit, Azienda Ospedaliera-Universitaria di Bologna, S. Orsola-Malpighi Hospital, Bologna, Italy; 4 Radiology Unit, Department of Diagnostic and Preventive Medicine, Azienda Ospedaliero-Universitaria di Bologna, S. Orsola-Malpighi Hospital, Bologna, Italy; 5 Medical Physics Unit, Radiology Unit, S. Orsola-Malpighi Hospital, Bologna, Italy; 6 Pathology Unit, Azienda Ospedaliero-Universitaria di Bologna, S. Orsola-Malpighi Hospital, Bologna, Italy; 7 Division of Anesthesiology, Azienda Ospedaliera-Universitaria di Bologna, S. Orsola-Malpighi Hospital, Bologna, Italy

**Keywords:** Hepatectomy, Liver failure, Hepatobiliary scintigraphy, SPECT, Mebrofenin

## Abstract

Hepatobiliary scintigraphy (HBS) has been demonstrated to predict post-hepatectomy liver failure (PHLF). However, existing cutoff values for future liver remnant function (FLR-F) were previously set according to the “50–50 criteria” PHLF definition. Methods of calculation and fields of application in liver surgery have changed in the meantime. The aim of this study was to demonstrate the role of HBS combined with single photon emission computed tomography (SPECT/CT) in predicting severity of PHLF, according to the International Study Group of Liver Surgery (ISGLS). All patients submitted to major hepatectomy with preoperative HBS-SPECT/CT between November 2016 and December 2019, were analyzed. Patients were resected according to hepatic volumetry. Receiver operating characteristic (ROC) curve analysis was performed to identify cutoffs of FLR function for predicting PHLF according to ISGLS definition and grading. Of the 38 patients enrolled, 26 were submitted to one-stage hepatectomy (living liver donors = 4) and 12 to two-stage procedures (portal vein embolization = 4, ALPPS = 8). Overall, 18 patients developed PHLF according to ISGLS criteria: 12 of grade A (no change in the patient’s clinical management) and 6 of grade B (change in clinical management). ROC analysis established increasingly higher cutoffs of FLR-F for predicting PHLF according to the “50–50 criteria”, ISGLS grade B and ISGLS grade A/B, respectively. HBS with SPECT/CT may help to assess severity of PHLF following major hepatectomy. Prospective multicenter trials are needed to confirm the effective role of HBS-SPECT/CT in liver surgery.

**Electronic supplementary material:**

The online version of this article (10.1007/s13304-020-00907-2) contains supplementary material, which is available to authorized users.

## Introduction

Post-hepatectomy liver failure (PHLF) represents one of the most feared complications by liver surgeons. PHLF occurs when the future liver remnant (FLR) is inadequate in sustaining its regenerative capacity in the postoperative course. At present, FLR volumetry is the standard method for determining whether a patient could be submitted safely to major hepatectomy [1]. However, Tc-99m mebrofenin hepatobiliary scintigraphy (HBS) has been also demonstrated to well predict PHLF and liver-related mortality before extended resections [2]. HBS has the advantage compared to other dynamic functional test, such as indocyanine green clearance (ICG) test, to measure not only the global liver function but also to take into account regional variations that may occur within the liver. For this reason, HBS has been described in major hepatectomy as well as in liver regeneration techniques, such as portal vein embolization (PVE) [3], Associating Liver Partition and Portal vein ligation for Staged hepatectomy (ALPPS) [4] and more recently in liver transplantation (LT) setting [5]. The most used cutoff value of FLR function (FLR-F) for predicting PHLF (2.69%/min/m^2^) was previously set using single-head gamma cameras (i.e. acquiring only the anterior planar projection of the liver) and according to the “50–50 criteria” (PT < 50% and serum bilirubin > 50 μmol/L on postoperative day 5) [6]. Thanks to the advent of dual-head gamma cameras (i.e. acquiring both anterior and posterior views of the liver) and SPECT/CT which is able to provide a 3-dimensional and more accurate measurement of postoperative remnant liver function [7], new considerations have to be made when using HBS-SPECT/CT in liver surgery. Furthermore, since the International Study Group of Liver Surgery (ISGLS) criteria to define PHLF [8] have been demonstrated to better perform than “50–50 criteria” in assessing severity of PHLF and are currently one of the most widely used criteria in clinical studies, the need to re-assess functional cutoffs to determine an adequate liver function is urgent.

The present study aims to assess the value of HBS combined with SPECT/CT in assessing PHLF according to ISGLS criteria.

## Methods

Between November 2016 and December 2019, patients submitted to major hepatectomy (defined as the removal of 3 or more continuous Couinaud segments) at Sant’Orsola-Malpighi Hospital (Bologna, Italy) were analyzed. During the study period, the volumetric assessment was systematically performed and used as the standard criterion to confirm the indication to surgery. Patients who could not undergo HBS-SPECT/CT were excluded from the present study. Patients with elevated (≥ 2.9 mg/dl) serum total bilirubin level were also excluded [9].

**Fig. 1:**
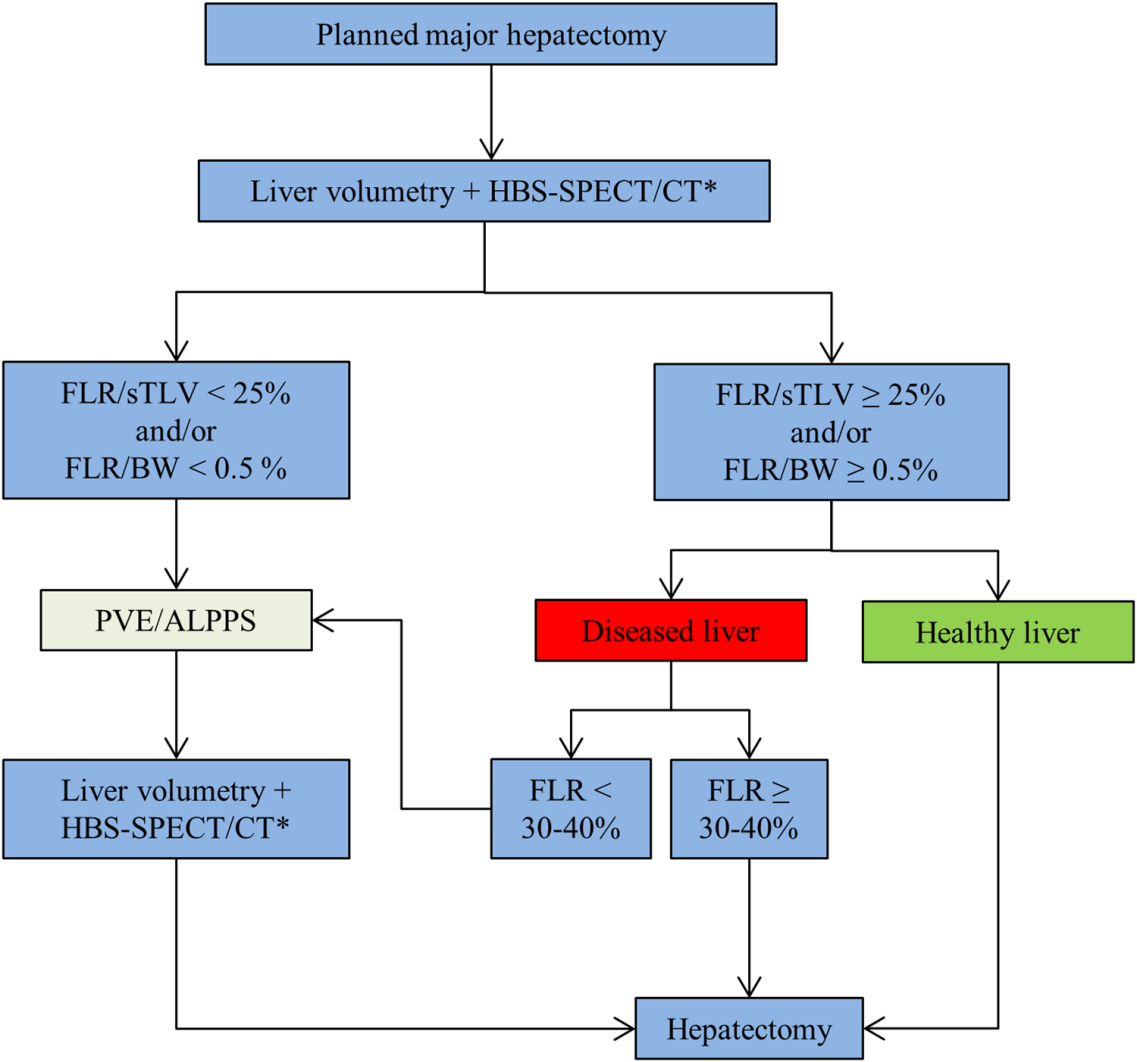
Algorithm of the study protocol. *HBS-SPECT/CT *hepatobiliary scintigraphy combined with single photon emission computed tomography, *FLR *future liver remnant, *sTLV *standardized total liver volume, *BW *body weight, *PVE *portal vein embolization, *ALPPS *associating liver partition and portal vein ligation for staged hepatectomy; * HBS-SPECT/CT was not taken into consideration in the decision process

In healthy livers, standardized future liver remnant ≥ 25% [14] or FLR/body weight (BW) ≥ 0.5% [10] was considered adequate to proceed with one-stage hepatectomy. Higher cut-offs were used in living liver donors (≥ 30%), in the presence of cholestasis, cirrhosis or in patients receiving prolonged use of chemotherapy. Techniques to induce FLR hypertrophy, such as PVE or ALPPS, were considered if FLR volume was deemed as inadequate. In two-stage procedures, HBS-SPECT/CT together with CT volumetry was always performed before the completion of the second stage of surgery (Fig. 1).

Data of single patients were prospectively collected into electronic spreadsheets. The study protocol conforms to the ethical guidelines of the 1975 Declaration of Helsinki (6th revision, 2008) as reflected in a priori approval by the institution's human research committee. Informed consent was obtained for all patients and the Institutional review board gave ethical approval to perform this study (SPECT-HR-17-01 n°130/2017/O/Oss).

### Variables

The main outcome of this study was PHLF according to ISGLS definition and severity grading [8]. Grade A PHLF represents a postoperative deterioration of liver function that does not require a change in the patient’s clinical management. Grade B PHLF requires a deviation from the normal postoperative course, but it can be managed without invasive treatment. Patients who develop grade C PHLF require an invasive procedure. Complications were classified according to the Dindo–Clavien classification of surgical complications [11] and major morbidity was defined as every complication ≥ grade 3A. Any death occurring during the postoperative 90-day period was considered a 90-day mortality. Data on patient demographics, tumor type, chemotherapy and procedure details were prospectively recorded.

### Liver volumetry

Liver volumes were assessed using cross-sectional imaging using portal phase CT or MRI. Volumetric reconstructions were performed by a single experienced radiologist (G.P.). Standardized future liver remnant was calculated as the ratio (%) between the FLR and the standardized total liver volume (sTLV), according to the Vauthey formula [12]: − 794.41 + 1267.28 × body surface area [13] (BSA) (m^2^). FLR/BW was calculated as the ratio (%) between FLR volume and patient’s body weight (BW), assuming a mean physical liver density of 1.00 g/mL [14]. Measured total liver volume (mTLV) was calculated after subtracting the tumor volume.

### Hepatobiliary scintigraphy and SPECT/CT

Briefly, patients were in supine position, with a large field-of-view (FOV) SPECT-camera (Discovery NM/CT 670 ES, GE Healthcare, Milan, Italy) over the liver and heart region. The suprasternal notch and the navel were used as landmarks for the superior and inferior edges of the FOV. First, a dual-head dynamic acquisition (36 frames of 10 s/frame, 128 matrix) was obtained immediately after the intravenous administration of 200 MBq Tc-99m mebrofenin (Bridatec, GE Healthcare, Milan, Italy). Mebrofenin is an iminodiacetic acid analogue that circulates in an albumin-bound form, taken up by hepatocytes and directly excreted into the bile canaliculi without undergoing any biotransformation [15].

**Fig. 2:**
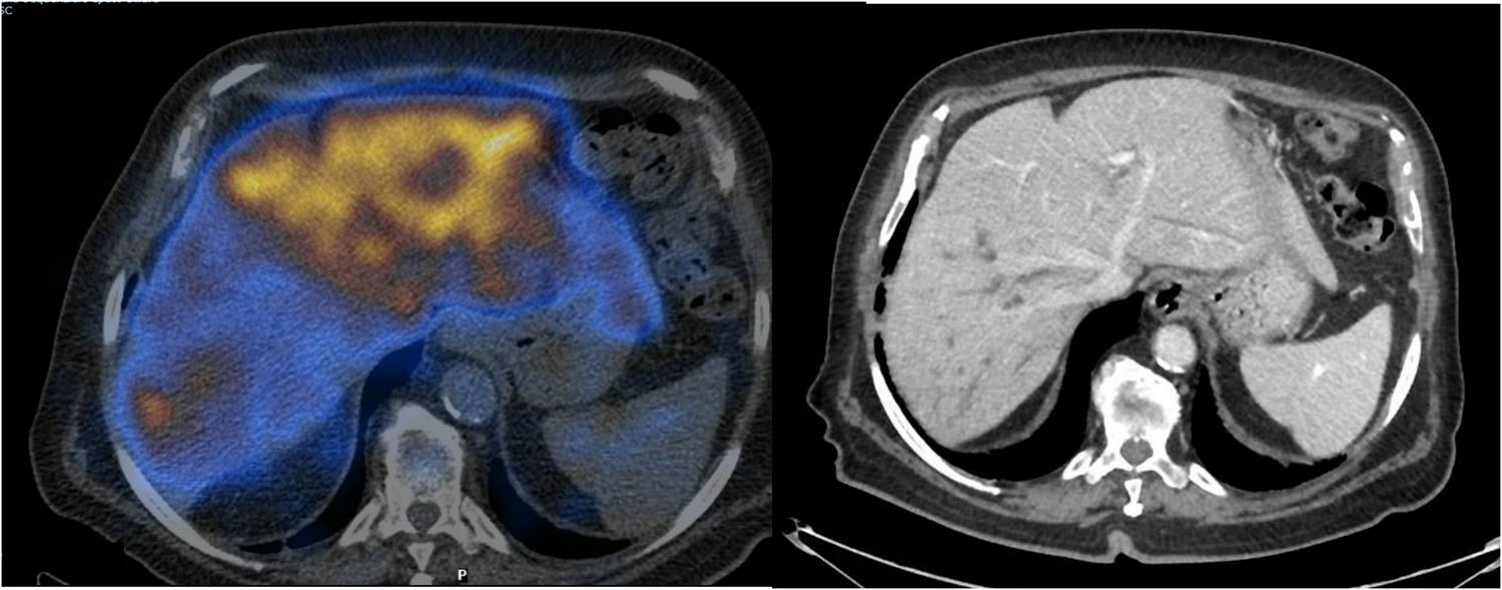
SPECT showing the distribution of function within the liver (**a**). The tumor, occupying entirely the right lobe, transferred the liver function almost to the left side (i.e. the future liver remnant). Abdominal computed tomography of the same patient (**b**)

The radiopharmaceutical was always prepared on-site the same day of injection and patients were required to fast 4 h before the scan [16]. Of note, the arm of the patient was placed perpendicular to the body and elevated at 25°–30° to prevent venous retention of the injected activity. After the first dynamic phase (360 s), the arms were immediately positioned above the head and a fast SPECT acquisition was then performed (60 projections of 5 s/projection, 128 matrix). This occurs on the peak of the hepatic time–activity curve, i.e. when the highest amount of the tracer is accumulated in the liver, before its excretion into the bile ducts, making it possible to depict the three-dimensional distribution of liver function (Fig. 2). A low-dose, non-contrast-enhanced CT scan was acquired for attenuation correction and anatomic mapping.

### Processing of images

**Fig. 3:**
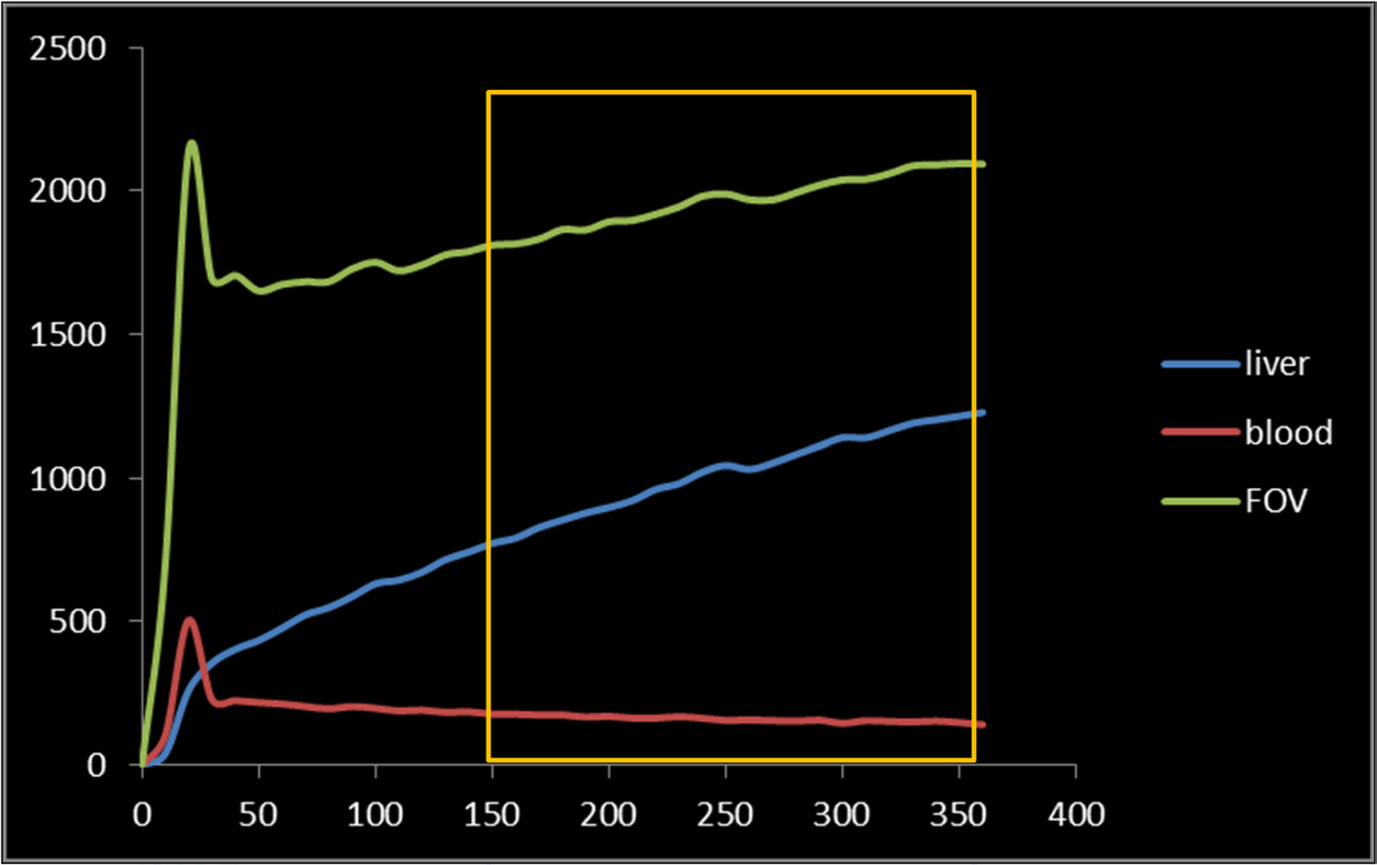
Time-activity curves calculated from three different regions of interest (ROI). Global liver function is measured using values obtained between 150 and 350 s post-injection according to Ekman’s formula [18]

Scintigraphic images were processed using a freely downloadable image analysis software package (Image J, https://imagej.nih.gov/ij/). Regions of interest (ROI) were manually drawn by the same experienced operator (C.P.) around the total liver, the heart/large vessels (serving as blood pool), and the total field of view (FOV). Analysis on dynamic images was done by drawing ROIs separately for anterior and posterior projections and calculating Geometric mean (Gmean) using the formula = $\sqrt {{\text{anterior}} \times {\text{posterior }}}$ for total liver uptake (TL-U) and by drawing ROIs on a single Gmean image (pixel-pixel) for total liver function (TL-F), as previously described [17]. From these ROIs, three time–activity curves were generated (Fig. 3). Calculations of TL-F (%/min) or TL-U (%) were performed using measured values obtained between 150 and 350 s post injection, i.e. during a phase of homogenous distribution of the agent in the blood pool before the phase of hepatic excretion [18, 19].

Separately, volumes of interest (VOIs) around the FLR and the total liver were manually outlined, using a contrast-enhanced CT scan linked to the SPECT images as a reference. Extrahepatic bile duct was not included in the liver VOIs. FLR-C (i.e. the 3-dimensional distribution of function within the FLR) was calculated dividing the counts (radioactivity) within the FLR’s VOI by the total counts within the entire liver’s VOI.

HIBA index (HIBA-*i*) and FLR-F both represent two alternative methods to calculate remnant liver function. FLR-F is the most extensively used scintigraphic index, developed at the Amsterdam Medical Center (AMC, Amsterdam, The Netherlands) and calculated by multiplying TL-F by FLR-C, as previously described [20]. HIBA-*i* is a novel measurement of remnant liver function described by the group of the Hospital Italiano de Buenos Aires (HIBA, Buenos Aires, Argentina) to predict PHLF in ALPPS and calculated as TL-U multiplied by FLR-C. Body surface area (BSA) was not retained for calculation of HIBA-*i* [19].

### Statistical analysis

Data were expressed in median and interquartile range (IQR). Differences between continuous variables were explored by the Mann–Whitney *U* test. Chi-squared test or Fisher's exact test was used for comparisons of categorical variables. Correlation between variables was tested using the Pearson correlation coefficient *r*. Receiver operating curve (ROC) analysis was undertaken to identify a cutoff value for predicting PHLF. Corresponding area under the curve (AUC), sensitivity, specificity, positive predictive value (PPV), negative predictive value (NPV), positive (LR +) and negative (LR–) likelihood ratios were calculated. The cut-off values were determined by seeking the largest sum of the sensitivity and specificity values, while maintaining the lowest likelihood ratio of a negative test and the highest likelihood ratio of a positive test. All statistical tests were two-tailed, and differences were considered significant at a *p*-value of ≤ 0.05. Statistical analysis was performed with SPSS Version 20.0 software (SPSS, Chicago, IL).

## Results

Overall, 38 patients were enrolled in the study, according to inclusion criteria. There were 23 males and 15 females. Median age was 64 years (range 31–82). Major morbidity was 18.4% whereas 90-day mortality was nil. At final pathology, underlying liver disease was found in 16 patients (cirrhosis in 4 patients and moderate to severe sinusoidal dilatation in 12 patients).

### One-stage hepatectomy

Twenty-two patients out of 38 (57.9%) with adequate FLR at CT volumetry were submitted to upfront major hepatectomy for hepatic neoplasms. There were 7 colorectal liver metastases (CRLM), 1 neuroendocrine liver metastasis, 3 intrahepatic cholangiocarcinomas (IHCC), 7 hepatocellular carcinomas (HCC), 1 gallbladder cancer, 1 perihilar cholangiocarcinomas (PHCC), and 2 hepatic cavernous hemangiomas. Preoperatively, median FLR/sTLV was 34.8% (IQR 30.6–63.4) and FLR/BW was 0.74 (IQR 0.63–1.36). Among them, right hepatectomy (RH) was carried out in in 12 patients (54.5%), left hepatectomy (LH) in 7, right trisectionectomy (RT) in 2 and left trisectionectomy (LT) in 1. PHLF occurred in 8 out of 22 patients and always after RH (4 of grade A and 4 of grade B). Subgroup analysis, including only RHs to make the two groups more homogenous in terms of remnant liver volumes, showed that only HIBA-*i* and FLR-F resulted significantly different between patients with and without PHLF (Table 1). Of note, TL-U and TL-F were comparable confirming a similar underlying global liver function in the two groups.

**Table 1:** Volumetric and functional parameters between patients submitted to one-stage right hepatectomy with and without PHLF according to ISGLS (all grades)

Variable	PHLF YES (*n* = 8)	PHLF NO (*n* = 4)	p-value
*Liver volumes*			
FLR, median (IQR), cc	487 (383–593)	591 (455–1053)	0.461
FLR/sTLV, median (IQR), %	30.2 (25.3–34.3)	47.1 (29.9–70.1)	0.214
FLR/BW, median (IQR), %	0.63 (0.51–0.72)	0.98 (0.64–1.49)	0.214
*Liver function*			
TL-F, median (IQR), %/min	10.7 (9–12.6)	11.76 (8.83–14.83)	0.683
TL-U, median (IQR), %	45.7 (43.3–52.1)	51.3 (46.6–59.5)	0.214
FLR-F, median (IQR), %/min/m^2^	1.50 (1.05–1.86)	3.80 (1.94–5.67)	0.016
HIBA-*i*, median (IQR), %	12.4 (8.9–18.1)	27.3 (18.3–40.7)	0.048

*PHLF *post-hepatectomy liver failure, *IQR *interquartile range, *FLR *future liver remnant, *sTLV *standardized total liver volume, *BW *body weight, *TL-F *total liver function, *TL-U *total liver uptake, *FLR-F *future liver remnant function, *HIBA-i *HIBA index

Four patients (10.5%) underwent HBS-SPECT/CT before living liver donation. According to CT volumetry, 3 LH and 1 RH were performed. Interestingly, in the only patient who underwent RH, despite a FLR volume of 30%, grade A PHLF occurred. After reviewing scintigraphic images, FLR-F and HIBA-*i* were 2.05%/min/m2 and 14.4%, respectively (Table 2).

**Table 2:** Characteristics of living donors evaluated with hepatobiliary scintigraphy

Patient	Age (yrs)	Sex	BMI (kg/m^2^)	FLR/sTLV (%)	FLR/BW (%)	HIBA-*i* (%)	FLR-F (%/min/m^2^)	Type of H	PHLF
1	59	F	23.5	29.6	0.64	14.4	2.05	RH	A
2	48	F	23.8	76.5	1.65	55	8.97	LH	–
3	46	M	24.4	55.9	1.23	41	5.52	LH	–
4	31	M	20.7	61.4	1.41	48.1	8.02	LH	–

*F* female, *M* male, *BMI* body mass index, *sTLV* standardized total liver volume, *BW* body weight, *HIBA-i* HIBA index, *FLR-F* future liver remnant function, *H* Hepatectomy, *RH* right hepatectomy, *LH* left hepatectomy, *PHLF* post-hepatectomy liver failure

### Two-stage procedures

Among all two-stage procedures (*n* = 12/38, 31.6%), preoperative PVE was performed in 4 cases and ALPPS in 8. In ALPPS group, partial parenchymal transection + PVE (“mini-ALPPS”) was performed in 4 out of 8 patients whereas PVL was chosen for portal vein occlusion (PVO) in 3 patients. After PVO, median FLR/sTLV was 30.9% (IQR 24.6–37.7) whereas FLR/BW was 0.63 (IQR 0.52–0.85). RH was carried out in 6 patients, RT in 5 and LT in 1.

Nine out of 12 patients developed PHLF (7 of grade A and 2 of grade B). In particular, 7 out of 8 patients submitted to ALPPS developed PHLF after completion of the second stage. HIBA-*i*, FLR-F and FLR-C resulted significantly different when comparing patients with and without PHLF, but not volumes (Table 3).

**Table 3:** Volumetric and functional parameters between patients submitted to two-stage procedures with and without PHLF according to ISGLS (all grades)

Variable	PHLF Yes (*n* = 9)	PHLF No (*n* = 3)	*p*-value
*Liver volumes*			
FLR/sTLV, median (IQR), %	27.1 (24–36.7)	36.4 (29.1–38.8)	0.282
FLR/BW, median (IQR), %	0.58 (0.50–0.83)	0.76 (0.58–0.82)	0.373
FLR/mTLV, median (IQR), %	30.6 (26.8–36.8)	44.1 (29.3–45.6)	0.282
*Liver function*			
TL-F, median (IQR), %/min	10.11 (8.03–12.02)	9.37 (7.44–10.53)	0.864
TL-U, median (IQR), %	50.9 (45.2–55.7)	48.6 (42.7–52.9)	0.864
FLR-C, median (IQR), %	35 (28.4–31.6)	69.4 (42–73.2)	0.036
FLR-F, median (IQR), %/min/m^2^	1.84 (1.50–2.34)	2.84 (2.49–3.11)	0.009
HIBA-*i*, median (IQR), %	17.8 (15.6–19.7)	29.6 (24–33.5)	0.009

*PHLF* post-hepatectomy liver failure, *IQR *interquartile range, *FLR *future liver remnant, sTLV standardized total liver volume, *BW *Body Weight, *mTLV *measured total liver volume, *TL-F *total liver function, *TL-U *total liver uptake; *FLR-C *counts within FLR/total counts, *FLR-F *future liver remnant function, *HIBA-i *HIBA index

**Fig. 4:**
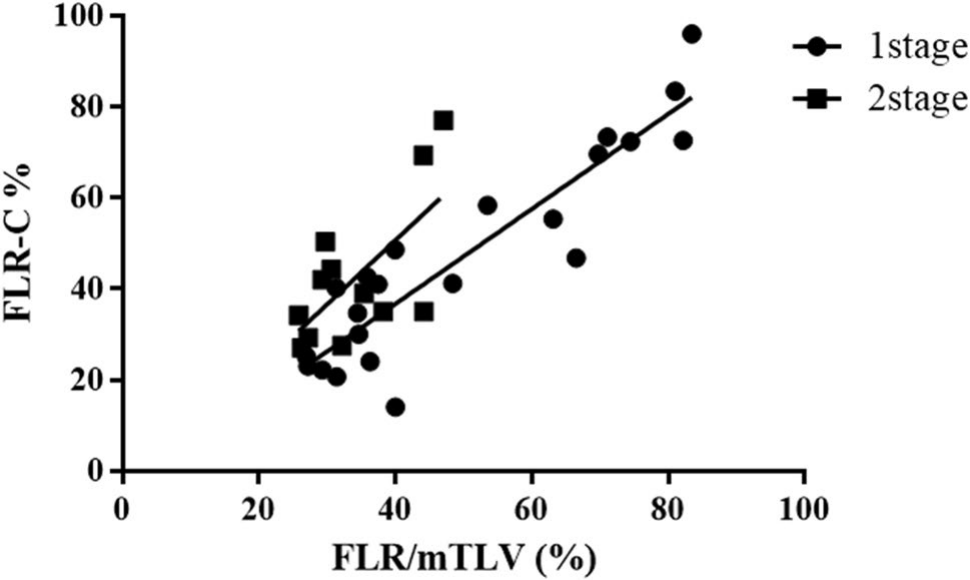
Correlation plot of distribution of liver volume versus function (FLR-C) within the future liver remnant (FLR) in one-stage (*r* = 0.912, *p* < 0.001) and two-stage hepatectomy (*r* = 0.662, *p* = 0.019). mTLV = measured total liver volume

Correlation between FLR-C and FLR/mTLV was 0.662 (*p* = 0.019) compared to one-stage hepatectomy when the same correlation increased to 0.912 (*p* < 0.001) (Fig. 4).

When SPECT/CT data were available for both pre- and post-portal vein occlusion (*n* = 10), median increase of FLR/sTLV was 49% (IQR 29.7–61) compared to that of FLR-C which was in median 39% (IQR 14.2–47.7). This difference changed when analyzing separately the increase in ALPPS (FLR/sTLV = 49% vs. FLR-C = 38%) and that observed after PVE (FLR/sTLV = 35% vs. FLR-C = 38%). Median interval between the first stage and CT volumetry was 17 days (IQR 7–29) compared to 19 days between the first stage and HBS-SPECT/CT (IQR 10–27).

### Safe cutoff of remnant liver function

**Fig. 5:**
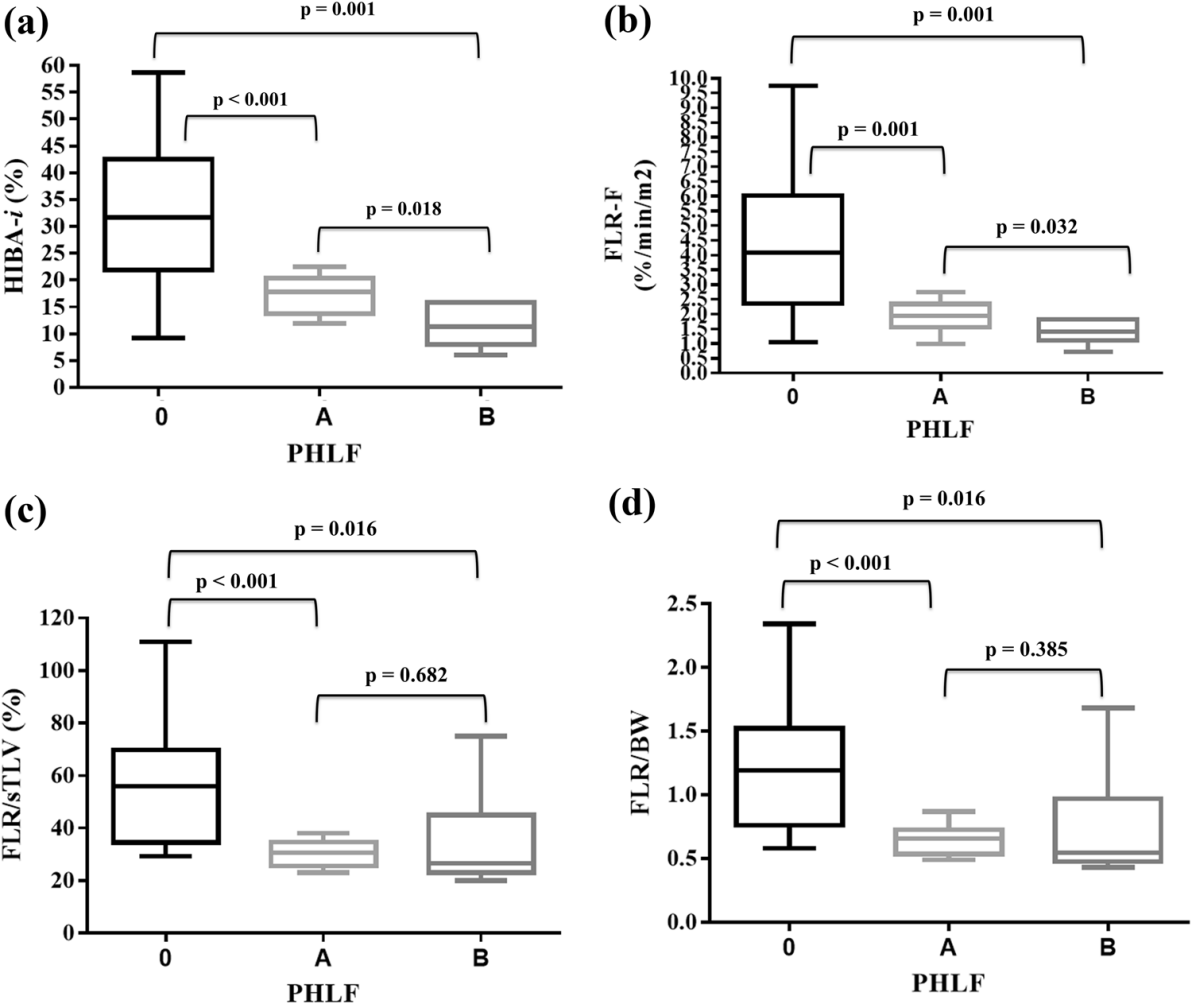
Box and whiskers plot showing the distribution of HIBA index (HIBA-*i*), FLR function (FLR-F), standardized future liver remnant (FLR/sTLV) and FLR/body weight (BW) between patients without post-hepatectomy liver failure (PHLF) and those with grade A or grade B PHLF. **a** Median HIBA-*i* was 31.6% (IQR 21.8–42.6) in patients without PHLF, 17.86% (IQR 13.8–20.4) in patients with grade A PHLF and 11.36% (IQR 8–15.9) with grade B PHLF. **b** Median FLR-F was 4.09%/min/m2 (IQR 2.36–6.01) in patients without PHLF, 1.96%/min/m^2^ (IQR 1.55–2.34) in patients with grade A PHLF and 1.40%/min/m2 (IQR 1.11–1.84) with grade B PHLF. **c** Median FLR/sTLV was 56% (IQR 34.3–70.2) in patients without PHLF, 30.9% (IQR 25.2–34.7) in patients with grade A PHLF and 26.4% (IQR 23.4–44.7) in patients with grade B PHLF. **d** Median FLR/BW was 1.19 (IQR 0.76–1.52) in patients without PHLF, 0.65% (IQR = 0.54–0.73) in patients with grade A PHLF and 0.54% (IQR 0.47–0.96) in grade B PHLF

Overall, eighteen patients developed PHLF according to ISGLS criteria: 12 of grade A (31.6%) and 6 of grade B (15.8%). Demographic and perioperative characteristics of patients with and without PHLF are summarized in Table 4. Four patients (10.5%) developed PHLF according to the “50–50 criteria”. Median sFLR and FLR/BW differed significantly between ISGLS grade 0 (no PHLF) and grade A or between grade 0 and grade B but they were not significantly different when comparing grade A and grade B PHLF (Fig. 5a–b). Conversely, HIBA-*i* and FLR-F were significantly different between grade 0 and grade A/B but also between grade A and grade B PHLF (Fig. 5c–d).

**Table 4:** Demographic and perioperative characteristics of patients with and without PHLF according to ISGLS criteria (all grades)

Variable	All patients (*n* = 38)	PHLF No (*n* = 20)	PHLF Yes (*n* = 18)
*Preoperative*			
Sex, F/M, *n*	15/23	8/12	7/11
Age, median (IQR), yrs	64 (54–71)	62 (48–68)	68 (58–74)
BMI, median (IQR), kg/m^2^	25.2 (23.7–27.4)	24.7 (22.4–27.2)	25.8 (23.8–27.8)
*ASA Score, n (%)*			
1–2	13 (34.2)	9 (45)	4 (10.5)
3–4	25 (65.8)	11 (55)	14 (89.5)
Neoadjuvant chemotherapy, *n* (%)	10 (26.3)	4 (20)	6 (33.3)
Oxaliplatin based, *n*	7	2	5
Irinotecan based, *n*	3	2	1
Preoperative PVE, *n* (%)	4 (10.5)	2 (10)	2 (11.1)
Total bilirubin, median (IQR), mg/dL	0.69 (0.52–0.94)	0.6 (0.38–0.88)	0.73 (0.56–1.85)
*Tumor type, n (%)*			
METS	13 (34.2)	5 (25)	8 (44.4)
HCC	8 (21)	5 (25)	3 (16.6)
IHCC	7 (18.4)	3 (15)	4 (22.2)
PHCC	3 (7.9)	1 (5)	2 (11.1)
Other	3 (7.9)	3 (15)	0
Living donors	4 (10.6)	3 (15)	1 (5.7)
*Intraoperative*			
*Type of liver resection*			
Right hepatectomy, *n* (%)	19 (50)	6 (30)	13 (72.2)
Right trisectionectomy, *n* (%)	7 (18.4)	3 (15)	4 (22.2)
Left hepatectomy, *n* (%)	10 (26.3)	10 (50)	0
Left trisectionectomy, *n* (%)	2 (5.3)	1 (5)	1 (5.6)
ALPPS, *n* (%)	8 (21)	1 (5)	7 (38.8)
Pringle maneuver, *n* (%)	29 (76.3)	17 (85)	12 (66.7)
Clamping time, median (IQR), min	28 (12–45)	36 (21–64)	15 (0–28)
*Postoperative*			
Hospital stay, median (IQR), days	10 (10–14)	8 (7–14)	12 (8–18)
Biliary leak, *n* (%)	6 (15.8)	2 (10)	4 (22.2)
Underlying liver disease, n (%)	16 (42.1)	7 (35)	9 (50)
Morbidity ≥ 3a^†^, *n* (%)	7 (18.4)	3 (15)	4 (22.2)
90-day mortality, *n* (%)	0	0	0

*PHLF *post-hepatectomy liver failure, *F *Female, *M *Male, *IQR *interquartile range, *BMI *body mass index, *ASA *American society of anesthesiologists, *PVE *portal vein embolization, *METS *metastases, *HCC *hepatocellular carcinoma, *IHCC *intrahepatic cholangiocarcinoma, *PHCC *perihilar cholangiocarcinoma, *ALPPS *associating liver partition and portal vein ligation for staged hepatectomy, ^†^according to Dindo-Clavien et al. [11]

**Fig. 6:**
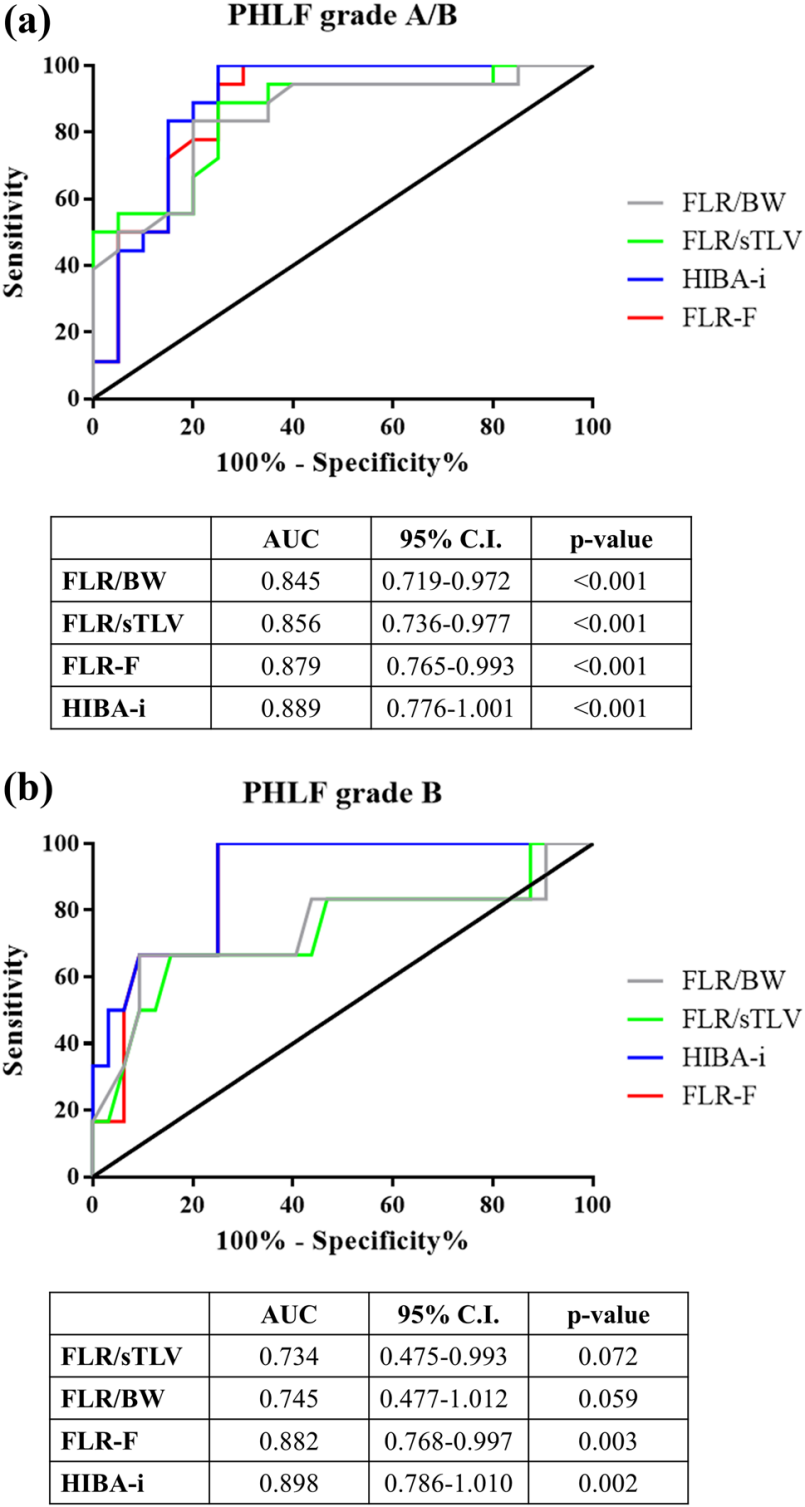
Receiver operator characteristics curve for future liver remnant function (FLR-F), HIBA index (HIBA-*i*), standardized FLR (FLR/sTLV) and FLR/body weight (BW) ratio in the diagnosis of post-hepatectomy liver failure grade A/B (**a**) and grade B (**b**), according to ISGLS criteria. *AUC *area under the curve, *CI *confidence interval

ROC analysis established a cutoff of 1.59%/min/m2 for FLR-F to predict PHLF according to the “50–50 criteria”**.** Higher cutoffs were identified to predict ISGLS grade B (1.85%/min/m2) and ISGLS grade A/B (2.79%/min/m2). Similarly, a cutoff of 15.9%, 16.8% and 23.8% was determined for HIBA-*i* (Table 5). The AUC of sFLR, FLR/BW, HIBA-*i* and FLR-F in predicting PHLF according to ISGLS grade B and ISGLS grade A/B PHLF were reported in Fig. 6a–b.

**Table 5:** Different functional parameters and their diagnostic accuracy in predicting PHLF

« 50–50»	Cutoff	AUC	95% CI	Se (%)	Sp	PPV	NPV	LR +	LR-
FLR-F, %/min/m^2^	1.59	0.90	0.80–0.99	100	88	50	100	8.50	0
HIBA-*i*, %	15.9	0.88	0.76–1.01	100	73	31	100	3.78	0

ISGLS grade B	Cutoff	AUC	95% CI	Se (%)	Sp	PPV	NPV	LR +	LR-
FLR-F, %/min/m^2^	1.85	0.88	0.77–0.99	100	75	43	100	4	0
HIBA-*i*, %	16.8	0.89	0.79–1.01	100	75	43	100	4	0

ISGLS grade A/B	Cutoff	AUC	95% CI	Se (%)	Sp	PPV	NPV	LR +	LR-
FLR-F, %/min/m^2^	2.79	0.88	0.76–0.99	100	70	75	100	3.33	0
HIBA-*i*, %	23.8	0.89	0.77–1.00	100	75	78	100	4	0

*PHLF p*ost-hepatectomy liver failure, *AUC *area under the curve, *CI *confidence interval, *Se *sensitivity, *Sp *specificity, *PPV *positive predictive value, *NPV *negative predictive value, *LR + * likelihood ratio positive, *LR–* likelihood ratio negative, *FLR-F *future liver remnant function, HIBA-*i *HIBA index

A cutoff of 1.85%/min/m2 (or 16.8% for HIBA-*i*) included 100% of grade B PHLF (*n* = 6) but also 41.7% of grade A PHLF (*n* = 5) and 15% of patients without PHLF (*n* = 3). When the cutoff increased to 2.79%/min/m^2^ (or 23.8% for HIBA-i), the remaining 58.3% of grade A PHLF (*n* = 7) and another 15% of patients without PHLF (*n* = 3) were included. Values higher than 2.79%/min/m^2^ (or 23.8% for HIBA-*i*) comprised only patients without PHLF (*n* = 14) (Figure S1a–b).

## Discussion

In this study, HBS combined with SPECT/CT was able to predict severity of PHLF according to ISLGS criteria. Consequently, results from the present study suggest to implement HBS-SPECT/CT in the standard preoperative workup of patients undergoing major hepatectomy to improve the profile of safety of surgery. Areas of interest are represented especially by one-stage hepatectomy with borderline FLR volumes, two-stage procedures and before living-donor hepatectomy. The ability of HBS-SPECT/CT in predicting the severity of PHLF, combined with the liver volumetry, may serve as a more accurate tool to decide whether or not to proceed with hepatectomy upon achievement of different cutoff values.

The correlation between liver volume and function is a highly debated topic which has recently regained interest within the HPB community due to the increasing spread of regeneration liver techniques [21]. For instance, despite rapid and impressive FLR hypertrophy and several studies demonstrating the potential role of ALPPS in overcoming limits of resectability [22], many concerns have been addressed to the safety of this procedure due to the high rate of liver-related mortality reported [10, 23, 24]. Indeed, despite the safest cutoff values of FLR volumes had been used, the incidence of PHLF was still reported to be high [25]. Hence, the importance of including a functional test in ALPPS as showed in our study, where all but one patient developed PHLF. Such a relative high incidence of PHLF was explained in ours as well in other previous studies, by the fact that the increase of liver volume in ALPPS cannot always be followed by a parallel increase in liver function [4, 19, 26]. In particular, this difference was more pronounced when comparing volumetric and functional increase in ALPPS vs. PVE, suggesting a more specific role of HBS-SPECT/CT for ALPPS surgery.

The correlation between volume and PHLF is controversial also before one-stage major hepatectomy [27]. In fact, since the presence of an underlying liver disease cannot always be assessed preoperatively with conventional tools as with sinusoidal dilatation, HBS-SPECT/CT is able, on the contrary, to estimate total and remnant liver function, thus helping liver volumetry to assess more precisely the risk of PHLF especially in borderline cases, such as before right hepatectomies, including right-lobe living-donor hepatectomies [28].

Measurement of liver uptake function by IODIDA clearance rate was first described in 1992 by Ekman et al. [18]. By applying this formula to Tc-99m mebrofenin clearance, after the preliminary report of Dinant et al. [29], De Graaf et al. established a cutoff value of 2.69%/min/m2 for residual liver function in a cohort of 50 patients submitted to major hepatectomy with a PHLF incidence of 16.4%, using the “50–50 criteria” definition [6]. Thereafter, the 2.69%/min/m^2^ value has been used, as a reference, in several other studies to decide whether or not to candidate patients to preoperative occlusion strategies [30–32], although method of calculations over the years was changed (by implementing SPECT and Gmean) [7] and different definitions of PHLF were used. Fifty-fifty criteria have been showed to predict more than 50% of mortality rate but they do not provide any classification of PHLF severity. Nowadays, the ISGLS criteria [8] are demonstrated to better perform than the “50–50 criteria” in predicting postoperative morbidity/mortality and are the most widely used criteria in clinical studies. A trend towards a longer hospital stay, a higher rate of major complications and mortality has been shown across different grades of PHLF severity [33].

We reported a different cutoff for PHLF when liver failure was defined according to “50–50 criteria” (1.59%/min/m^2^) whereas it approached the reference value of 2.69%/min/m^2^ only when including also grade A PHLF according to ISGLS criteria (2.79%/min/m^2^). Only few other studies have reported different cutoffs of FRL-F [34] (Table 6) whereas more recently, Tomassini et al. [35] have confirmed the 2.7%/min/m2 cutoff also in ALPPS surgery. Differently, the group of Buenos Aires established in 2017 a lower cutoff (1.72%/min/m^2^) to predict clinically significant PHLF in ALPPS interstage which was similar to our cutoff for ISGLS B PHLF (1.85%/min/m^2^) [19]. The same Authors also proposed an alternative measurement of remnant liver function called HIBA index which seemed to give an almost perfect diagnostic performance of PHLF using a cutoff of 15%. In our study, HIBA-*i* showed high diagnostic accuracy of PHLF not only in ALPPS but also before one-stage hepatectomy, suggesting a great potential for this novel index which, however, still needs to be validated in larger studies.

**Table 6:** Studies reporting calculated cut-offs of FLR-F

Author	Year	N pts	Type of liver surgery (%)	PHLF (%)	PHLF definition	Cutoff of FLR-F	Other findings
Tomassini et al. [35]	2020	98	ALPPS (100)	14	ISGLS (B/C)	2.7%/min/m^2^	Patients with KGR < 4.1%/day and FLR-F < 2.7%/min/m^2^ were at high risk of PHLF
Serenari et al. [19]	2018	20	ALPPS (100)	20	ISGLS (B/C), 50–50 or Peak > 7	1.69%/min/m^2^	HIBA-*i* = 15% had a high diagnostic performance in ALPPS interstage
Olthof et al. [9]	2017	116	OSH (93.1) PVE (6.9)	23.3	ISGLS (B/C)	8.5%/min	HBS showed higher predictive value when performed with bilirubin levels < 2.9 mg/dl
Chapelle et al. [34]	2015	88	OSH (100)	13.6	ISGLS (B/C)	2.3%/min/m^2^^*^	FLR-F was the only independent predictive factor for PHLF
De Graaf et al. [2]	2010	55	OSH (100)	16.4	50–50	2.69%/min/m^2^	HBS had better diagnostic accuracy compared to volume
Dinant et al. [29]	2007	46	OSH (100)	13	50–50	2.5%/min/m^2^	2.2%min/m2 for PHLF-related mortality

*PHLF *post-hepatectomy liver failure, *ISGLS *international study group of liver surgery, *KGR *kinetic growth rate, *OSH *one-stage hepatectomy, *ALPPS *associating liver partition and portal vein ligation for staged hepatectomy, *FLR-F *future liver remnant function, *HBS *hepatobiliary scintigraphy, *PVE *portal vein embolization, *TL-F *total liver function, *TLV *total liver volume, *MRI *magnetic resonance imaging

^*^Calculated as TL-F multiplied for FLR/TLV measured by MRI

In our study, when we compared patients with and without PHLF, FLR-F and HIBA-*i*, as well as liver volumes*,* resulted significantly lower in the PHLF group. More interestingly, these scintigraphic parameters resulted significantly different also when comparing grade A and grade B PHLF, but not volumes. This is in line with other reports showing a not clear association between FLR volumes and incidence of PHLF when ISGLS criteria were used [36]. ROC analysis established increasingly higher cutoffs of FLR-F to predict PHLF according to the “50–50 criteria”, ISGLS grade B and ISGLS grade A/B, respectively. This finding may be indicative of the potential value of HBS-SPECT/CT to predict a clinically significant liver failure or a liver failure which impacts the postoperative course to a lesser extent in terms of morbidity and mortality, using different cutoffs of liver function. If we look back at 2007, Dinant et al. [29] had already showed that a lower value of FLR-F (2.2%/min/m^2^) was able to predict liver-failure-related mortality compared to when only PHLF was analyzed (2.5%/min/m^2^).

Our finding may be clinically relevant: for instance, when the risk of PHLF must be reduced to zero, as in living-donor hepatectomy [5, 36, 37], the safest cutoff value of remnant liver function (FLR-F > 2.79%/min/m^2^ or HIBA-i > 23.8%) should be used. On the contrary, if the risk of dropout between stages, as in PVE or TSH, is deemed to be higher than the risk of grade A PHLF [22], a lower cutoff can be considered (FLR-F ≥ 1.85%/min/m^2^ or HIBA-*i* ≥ 16.8%). Nevertheless, we suggest to use a reference range with upper and lower reference limit of liver function (FLR-F = 1.9–2.8%/min/m^2^, HIBA-*i* = 17–23%) rather than a single cutoff value to predict PHLF. In fact, ISGLS A and B may overlap each other in some cases and moreover, liver function measurement can be susceptible to several bias, such as fasting [16], preparation of Tc-99m mebrofenin and images processing or liver function analysis [17, 38], whose differences could lead to altered results [39]. For instance, in our series, a ROI-ROI calculation was used to calculate HIBA-*i*, i.e. calculating Gmean by drawing ROIs separately for anterior and posterior projection, whereas a pixel–pixel calculation, i.e. drawing ROIs on a single Gmean image, was used to calculate FLR-F which is the current method of calculation at the AMC. FLR-F has been found to be significantly different, in particular smaller, when compared to a pixel–pixel calculation [40]. This would explain the difference found in previous reports for FLR-F [19] but at the same time confirming in our study a similar cutoff value of HIBA-*i* (16.8%) for predicting ISGLS B PHLF.

Our study has some limitations. First, we did not compare sensitivity and specificity using appropriate statistical tests; therefore, there is weak evidence, also due to the small number of patients included, that the diagnostic accuracy of HIBA-*i* was superior to that of FLR-F in predicting PHLF as well as of function over volume. However, the aim of our study was to show the role of HBS-SPECT/CT, regardless of the index used, in predicting PHLF, although this argument surely will require further dedicated research. In the meantime, at least in our opinion, volumetric and functional assessments should be both performed before major hepatectomy especially in right-sided hepatectomies, ALPPS or right-lobe living-donor hepatectomies.

Second, no patients experienced grade C PHLF and we could not provide any functional value for this severity grade but we would have expected lower values to those reported for grade B PHLF.

Last but not least, although one- and two-stage hepatectomies were put all together in this study, there is no reason to believe that cutoff of residual liver function should have been different if they had been analyzed separately. In fact, PHLF represents a unique entity for both one-stage and two-stage procedures, representing simply the epiphenomenon of a poor functional reserve which can be better assessed preoperatively by means of HBS-SPECT/CT.

## Conclusion

HBS combined with SPECT/CT seems a promising tool able to predict severity of PHLF especially in specific fields of liver surgery. Prospective multicenter trials will be needed to confirm our preliminary data and to define more precise cutoffs of minimal residual liver function.

## Electronic supplementary material

Below is the link to the electronic supplementary material.

Supplementary file1 (TIF 189 kb)

Supplementary file2 (TIF 186 kb)
