# Supplementary figures and images for: The role of hepatobiliary scintigraphy combined with spect/ct in predicting severity of liver failure before major hepatectomy: a single-center pilot study
Source: Updates Surg. 2020 Nov 2;73(1):197–208. doi: 10.1007/s13304-020-00907-2 (PMC7889556; doi:10.1007/s13304-020-00907-2)

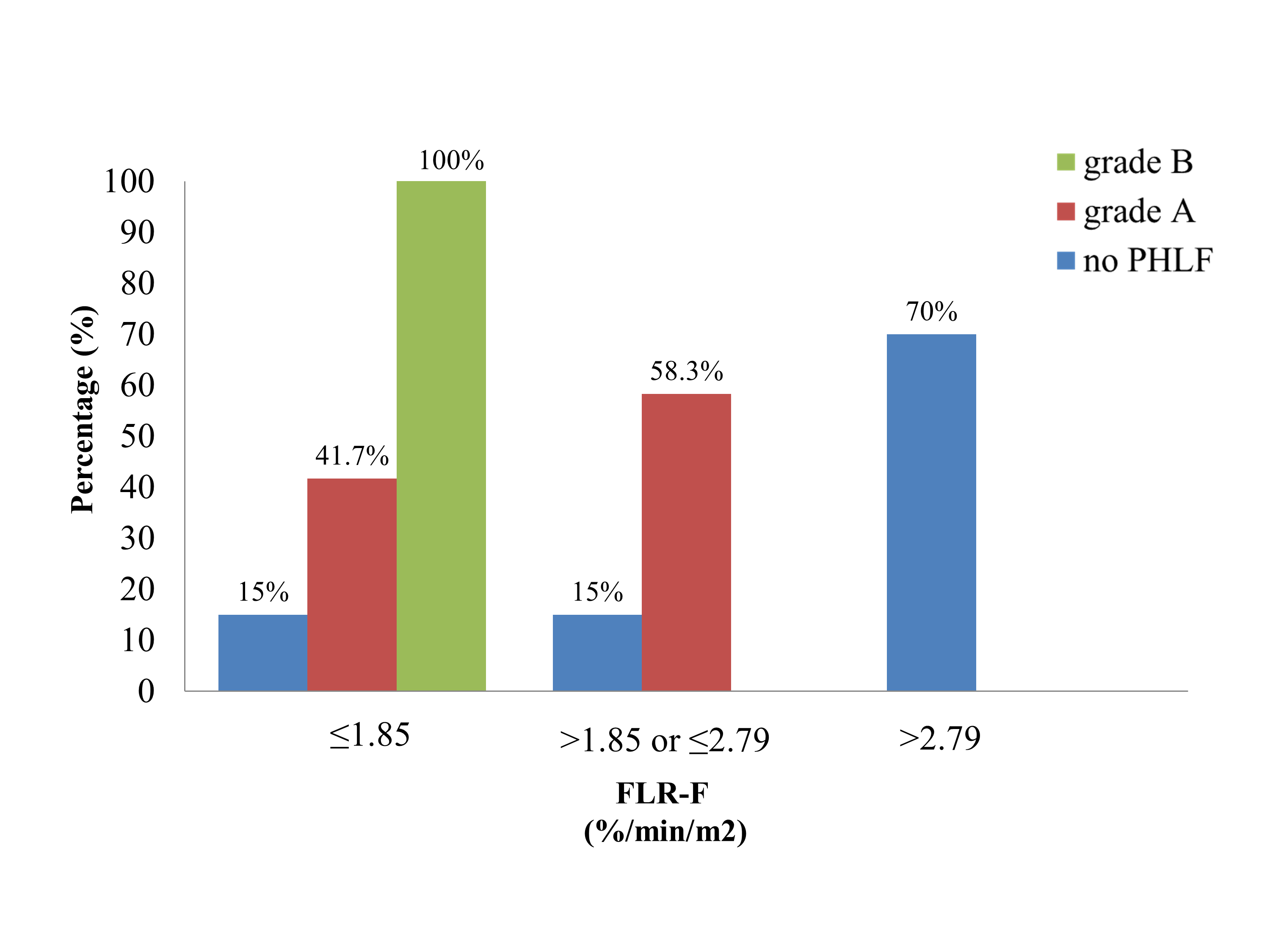

Supplement: Supplementary file 1 — Supplementary file1 (TIF 189 kb) [file 13304_2020_907_MOESM1_ESM.tif]

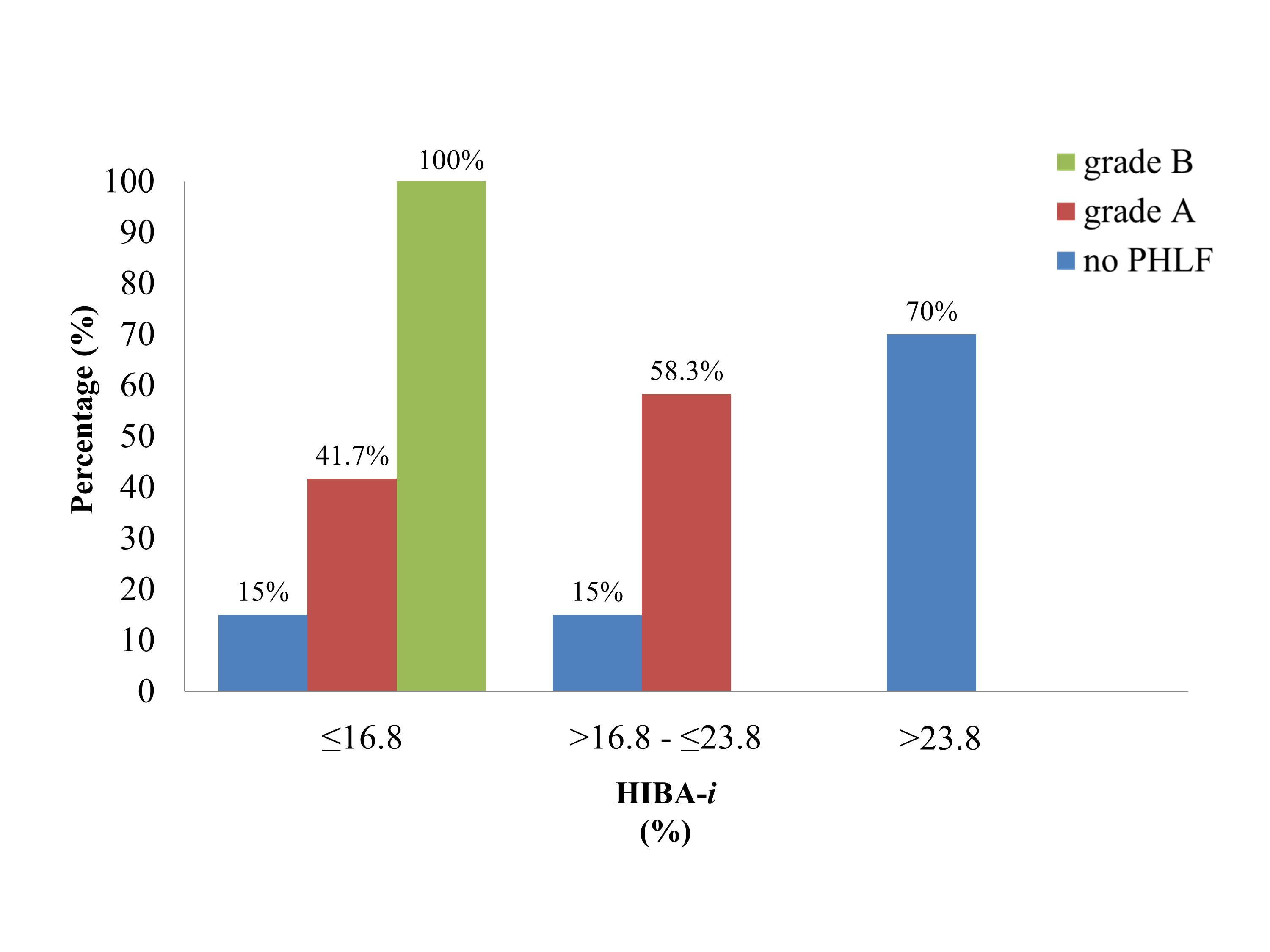

Supplement: Supplementary file 2 — Supplementary file2 (TIF 186 kb) [file 13304_2020_907_MOESM2_ESM.tif]
